# Reproductive history before and after HIV diagnosis: A cross-sectional study in HIV-positive women in Spain

**DOI:** 10.1097/MD.0000000000005991

**Published:** 2017-02-03

**Authors:** Victoria Hernando, Belen Alejos, Marta Montero, MªJesús Pérez-Elias, José Ramón Blanco, Livia Giner, Juan Luis Gómez-Sirvent, Jose Antonio Iribarren, Enrique Bernal, Francisco Bolumar

**Affiliations:** a Red de Investigación en Sida, Centro Nacional de Epidemiología, Instituto de Salud Carlos III; b CIBER de Epidemiología y Salud Pública (CIBERESP); c Hospital Universitario La Fe, Valencia; d Hospital Universitario Ramón y Cajal, Madrid; e Hospital Universitario San Pedro-CIBIR, Logroño; f Hospital Universitario de Alicante, Alicante; g Hospital Universitario de Canarias, Tenerife; h Hospital Universitario Donostia, Donostia; i Hospital Universitario Reina Sofia, Murcia; j Universidad de Alcalá, Alcalá de Henares, Madrid, Spain; k City University of New York School of Public Health at Hunter College, New York, USA.

**Keywords:** abortions, HIV infection, pregnancy

## Abstract

The aim of this study is to examine the reproductive history of human immunodeficiency virus (HIV)-positive women, before and after HIV diagnosis, to describe the characteristics of women with pregnancies after HIV diagnosis, and to assess the prevalence of mother-to-child transmission.

A cross-sectional study was performed among women within reproductive ages (18–49) selected from the cohort in the Spanish AIDS Research Network (CoRIS). A descriptive analysis of the pregnancy outcomes was made according to women's serostatus at the moment of pregnancy and association of women's characteristics with having pregnancy after HIV diagnosis was evaluated using logistic regression models.

Overall, 161 women were interviewed; of them, 86% had been pregnant at least once and 39% after HIV diagnosis. There were 347 pregnancies, 29% of them occurred after HIV diagnosis and in these, 20% were miscarriages and 29% were voluntary termination of pregnancy. There were 3 cases of mother-to-child transmission among the 56 children born from HIV-positive mothers; in these cases, women were diagnosed during delivery. Having a pregnancy after HIV diagnosis was more likely when the younger women were at the time of diagnosis: odds ratio (OR) = 1.29 (95% confidence interval 0.40–4.17) for 25 to 29 years old, OR = 0.59 (0.15–2.29) for 30 to 34 years old, OR = 0.14 (0.03–0.74) for ≥35 years old, compared with those <25 years at diagnosis, who were diagnosed for ≥5 years (OR = 5.27 [1.71–16.18]), who received antiretroviral treatment at some point (OR = 9.38 [1.09–80.45]), and who received information on reproductive health (OR = 4.32 [1.52–12.26]).

An important number of pregnancies occurred after HIV diagnosis, reflecting a desire for motherhood in these women. Reproductive and sexual health should be tackled in medical follow-ups.

## Abstract 2

Supplemental Digital Content is available in the text

## 1 Introduction

Around 46% of the people living with human immunodeficiency virus (HIV) worldwide are women.^[1]^ In Spain, during the period 2008 to 2013 approximately 18% of the new HIV diagnoses were in women.^[2]^ Most of the diagnoses in women happened between the ages of 25 to 39 years old, which within a women's reproductive age, making imperative to address in these patients some gender-specific issues, such as reproductive desire, contraceptive use, and pregnancy. The detection of an HIV infection may have a significant impact on women's decision on whether or not to have children and on whether to continue or to terminate a pregnancy, but few studies have addressed this issue in depth.^[3–5]^

In the early years of the epidemic, mother-to-child transmission (MTCT) was a major route of HIV acquisition as there was a high risk of infection for the newborn, but with the introduction of antiretroviral therapy vertical transmission diminished dramatically, and in the period from 2008 to 2013 only 49 cases of MTCT occurred in Spain.^[2]^ Still, the fear of transmitting HIV to the newborn continues to influence HIV-positive women when thinking about childbearing, even when the improved life expectancy and quality of life in HIV-positive patients make having children a more popular option.^[2,4,6–8]^

The main objective of this study was to explore the reproductive history of HIV-positive women, before and after HIV diagnosis, to describe the characteristics of women with pregnancies after HIV diagnosis, and to assess the prevalence of MTCT.

## 2 Methods

### 2.1 Design, simple frame, and subjects

A cross-sectional observational study was designed within the cohort of HIV-positive adults in the AIDS Research Network (CoRIS). CoRIS is a prospective, open, and multicenter cohort of patients newly diagnosed with HIV naïve to antiretroviral treatment at cohort entry of 28 centers participating from 13 Autonomous Regions of Spain.^[9,10]^ Nine hospitals and one sexually transmitted disease clinic in each of 8 cities (Madrid, Valencia, Logroño, Santa Cruz de Tenerife, Elche, Alicante, Murcia, and Donostia) from 6 Autonomous Communities participated in this study (see Annex 1). The study was approved by the Ethics Committee of the Institute of Health Carlos III (approved in 2012) and each patient signed an informed consent form.

In the first phase of the study, all women between 18 and 49 years old in the CoRIS cohort in all the participating settings were included until January 2010. A healthcare provider in each participating center contacted the selected women to explain the project and to request their participation and written consent. When the coordinating center received the signed consent, in the second phase a person from the research team contacted each woman by phone to conduct the interview. Among 494 women selected in the first phase of the study, 240 (48%) could not be located or no longer had follow-ups in the hospital or clinic. Of the remaining 254, 63 (25%) refused, 36 (14%) were excluded for different reasons (not speaking Spanish, not having a telephone, living on the street, or being in prison) and 155 (61%) agreed to participate. One of the hospitals participating started to recruit subjects when the fieldwork was almost completed, and because of that, only 6 women from this hospital were included. Finally, a total of 161 women were interviewed between November 2011 and December 2012.

### 2.2 Definition of variables

A structured ad hoc questionnaire (see Annex 2, supplemental content) was designed and administered through telephone interviews by 2 trained researchers between November 2011 and April 2012. Information on sociodemographic and clinical characteristics, and sexual and reproductive history was collected.

We included, among other sociodemographic variables: country of origin (categorized as “Spain” or “Other countries” which was subsequently categorized to “Europe,” “Latin America,” or “Africa”), educational level (“low” for no formal education or only primary education, “medium” for complete secondary education, and “high” for university degree), and current occupational status (“employed,” “unemployed,” or “other”—which included students, homemakers, and pensioners).

Clinical variables comprised: HIV transmission category (injecting drug user [IDU]/heterosexual contact/other), hepatitis C virus (HCV) (positive/negative), AIDS defining conditions (Yes/No), time since HIV diagnosis (“<5,” “5–10,” and “>10” years), and nadir CD4 count (“<200 CD4 cell/mm^3^” or “200 or more CD4 cell/mm^3^”).

Data about current partners and cohabitation status were also gathered and grouped into “has a stable partner and they live together,” “has a stable partner but they do not live together,” and “no stable partner.” Women were asked about the total number of sexual partners in the last 12 months as well as the type of sexual partner (stable or occasional) and whether they had unprotected sexual intercourse.

Women were asked to state if they had looked for or received information on sexual and reproductive health and whether they had disclosed their HIV infection to their closest relatives and friends. Social support was evaluated through a 4-item MOS-SSS version, with a range going from 1 (none of the time) to 5 (all of the time) for each item. “Social support” was classified as “Low” when the score was below 12 points and “Medium” if the score was 12 points or higher.

The main outcomes of this analysis were about pregnancies and their results. So, “pregnancy outcomes” were defined as “live birth” if the pregnancy was carried to term and the newborn lived at least 7 days, “voluntary termination of pregnancy (VTP)” if woman decided not to continue the pregnancy and “miscarriage” if it was a spontaneous loss of the fetus. We also collected motivations for the VTP (“Financial issues,” “Professional/work-related issues,” “Personal issues,” “Health issue,” “Active consumption of drugs,” and “Do not know/Do not answer”), the existence of a premature birth (labor occurred prior to 37 weeks of pregnancy or before), and type of labor (vaginal/caesarean section). The newborn's HIV diagnosis and antiretroviral treatment were also reported.

Data were analyzed according to the moment of the pregnancy in relation to HIV diagnosis: “Before HIV diagnosis” if the pregnancy had occurred before HIV diagnosis and “After HIV diagnosis” if the pregnancy had occurred after HIV diagnosis or HIV diagnosis occurred during pregnancy or delivery.

Finally, we compared the characteristics of women who had been pregnant after HIV diagnosis with those women who had been pregnant only before their HIV diagnosis, who had never been pregnant or who had discovered their HIV diagnosis during pregnancy or delivery. In the group of women who had been pregnant after HIV diagnosis were included those women who had had pregnancies before and after their HIV diagnosis.

This analysis was rerun excluding those women who had never been pregnant as sensitivity analysis (data not shown).

### 2.3 Statistical analysis

Pregnancies’ characteristics were described according to women's serostatus using frequency tables for categorical variables and median and interquartile range (IQR) for continuous ones. The *P* values were estimated using robust methods that assumed correlations between pregnancies which occurred in the same woman and independence among women recruited.

Multivariable logistic regression models were fitted to evaluate association of women's characteristics with having pregnancy after HIV diagnosis. Crude and adjusted odds ratios (ORs) with their 95% confidence intervals (95% CIs) were obtained as the measure of association.

All statistical analyses were performed using Stata software (Version 13.0, College Station, TX).

## 3 Results

### 3.1 Characteristics of the participants

Table 1 shows the characteristics of women at the time of the interview.

**Table 1:**
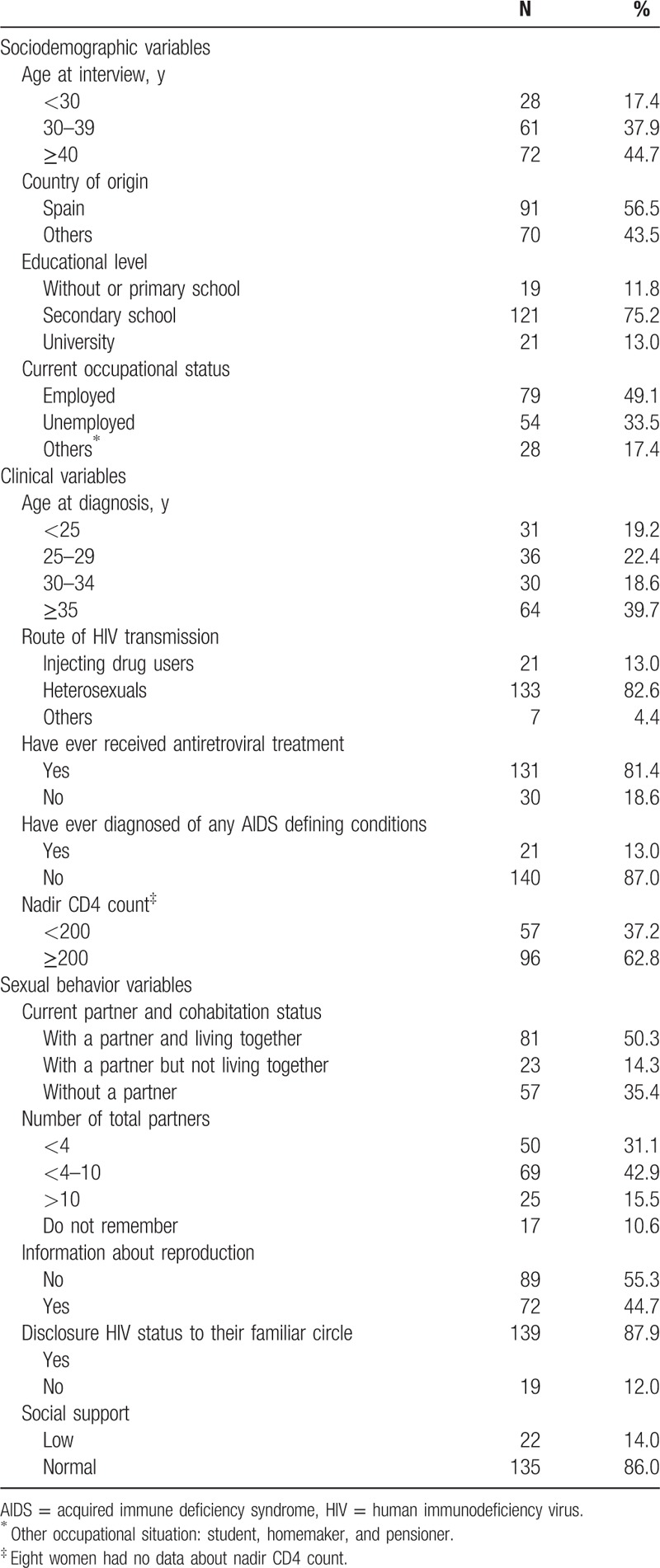
Sociodemographic, sexual behavior, and clinical characteristics of 161 women interviewed.

The median age of the 161 women at the time of the interview was 38 years (IQR 32–44). Spanish origin accounted for 56.5% (n = 91) while the remaining 43.5% was divided into: 48.6% (n = 34) from Latin America, 27.1% (n = 19) from Europe (10 from Russia), 24.3% (n = 17) from Africa, and 7 from Equatorial Guinea. The median time of residence in Spain of these non-Spanish women was 8 years (IQR 4–11).

Most of the women interviewed (75.2%) had secondary education and nearly half (49.1%) worked outside their home at the time of the interview. Women with a partner accounted for 64.6% (n = 104), all male, and 81 (77.8%) cohabited at the time of the interview. Of those partners, 41.3% (n = 43) were also diagnosed with HIV and only 1 partner had an unknown HIV serostatus.

Women had been diagnosed in median 4 years (IQR 2–7) before the interview. Unprotected sex was the main route of HIV acquisition (82.6%, n = 133) while 13.0% (n = 21) were injecting drug users. Around 13.0% (n = 21) had suffered an AIDS defining illness and 81.4% (n = 131) had received antiretroviral treatment at some point. HCV infection was found in 17.4% (n = 28).

### 3.2 Reproductive history

Of the 161 interviewed women, 14.3% (n = 23) had never been pregnant, 52.2% (n = 84) had gone through 1 or 2 pregnancies, 22.4% (n = 36) had 3 or 4 pregnancies, and 11.2% (n = 18) had 5 or more pregnancies (data not shown). Regarding HIV serostatus, of the 138 women who became pregnant, only 75 become pregnant before HIV diagnosis, only 24 after HIV diagnosis, and 39 before and after HIV diagnosis. The percentage of nulliparous women was 31.0% (n = 50), 57.1% (n = 92) had 1 or 2 children, and 11.8% (n = 19) had 3 or more children. This gave a total of 347 pregnancies and 201 children born.

Table 2 shows the characteristics of the 347 pregnancies according to women's HIV serostatus.

**Table 2:**
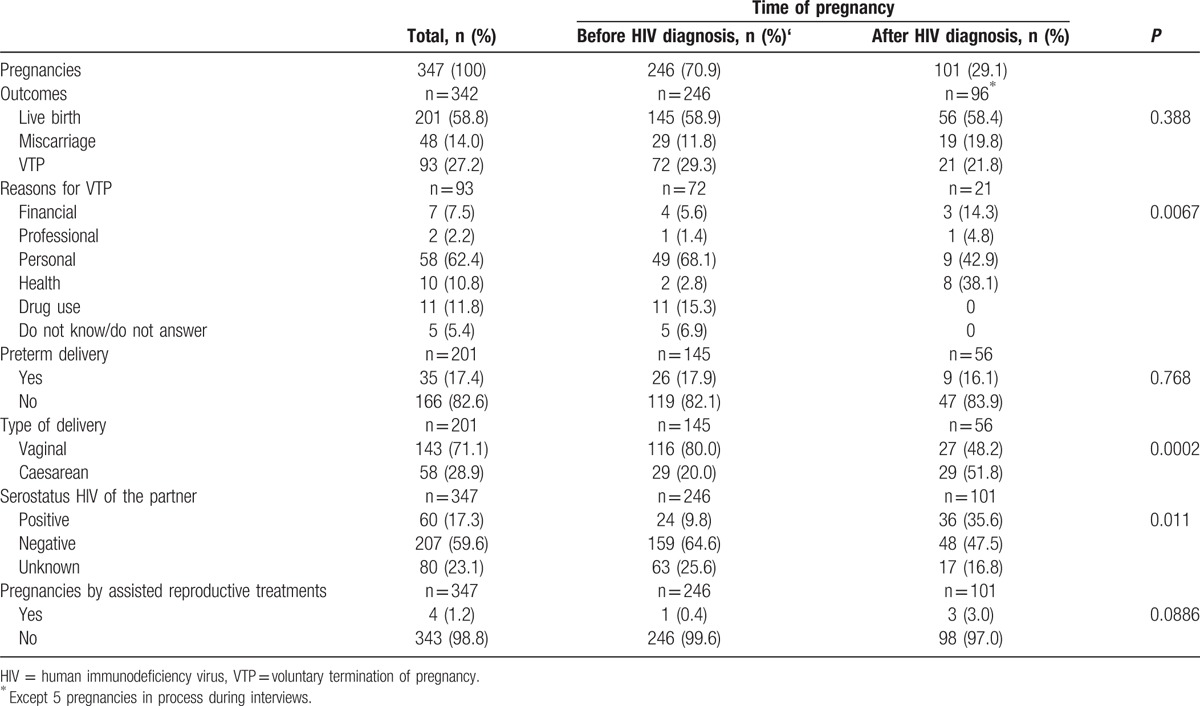
Characteristics of 347 pregnancies according to HIV serostatus of women.

Of the 347 pregnancies (138 women), 246 (70.9%) (65 women) occurred before the women were diagnosed with HIV and the remaining 101 (29.1%) afterward. These 101 pregnancies occurred among 63 women. At the time of the interview 5 women were pregnant. The median time between HIV diagnosis and the first pregnancy after HIV diagnosis was 2 years (IQR 1–4 years), excluding pregnancies with a diagnosis during antenatal care.

No statistically significant differences were observed in the percentage of pregnancies to full term before and after HIV diagnosis in women (58.9% vs 58.4%, respectively; *P* = 0.937), or regarding the number of preterm births (17.9% vs 16.1%, *P* = 0.755), but a significant increase in the percentage of cesarean deliveries, 20.0% to 51.8% (*P* < 0.001) was found.

In pre-HIV diagnosis pregnancies, 9.7% (n = 24) of them, women knew their male partners to be HIV-positive, while 25.5% (n = 63) did not know the serostatus of their partners. In post-HIV pregnancies, 36.0% (n = 36) occurred with a seroconcordant partner, 47.0% (n = 47) with a discordant one and in 17.0% (n = 17) the serostatus of the partner was unknown.

### 3.3 Miscarriages

Of the 138 women who had been pregnant at least once in their life, 18.1% (n = 25) had 1 or 2 miscarriages and 5.1% (n = 7) 3 or more. Out of the 347 recorded pregnancies, 14.0% (n = 48) ended in spontaneous miscarriage increasing to 19.8% (n = 19) when taking into consideration only pregnancies after diagnosis (*P* = 0.092).

### 3.4 Voluntary termination of pregnancy

Of the 138 women with pregnancies, 36.2% (n = 50) had undergone 1 or 2 VTPs and 5.7% (n = 8) 3 or more, leading to 27.2% (n = 93) of all pregnancies ending in VTP. In pregnancies occurring before the diagnosis of HIV, 29.3% (n = 72) were VTP in contrast to 21.8% (n = 21) in pregnancies after diagnosis (*P* = 0.239).

The reasons given by women to undergo VTP varied depending on whether the pregnancy occurred before or after the diagnosis of HIV (*P* < 0.001). The main reason for women to end a pregnancy before HIV diagnosis was “Personal issues” (68.1%, n = 49), a category that included being too young, lacking a stable partner, interference with studies/career, or not wanting children at that moment. The next most selected reason, with 15.3% (n = 11), was the “Active consumption of drugs” at the time of the pregnancy, and other motives stated were “Financial issues” 5.6% (n = 4), “Health issues” 2.8% (n = 2), “Professional/work-related issues” 1.4% (n = 1). Finally, 6.9% (n = 5) did not disclose a reason or were unwilling to answer this question.

Regarding the reasons claimed by women after HIV diagnosis, “Personal issues” remained the most important with a 42.9% (n = 9) although the reasons under this category were slightly different from the previous group: not wanting children and short-term partner or problematic relationship. “Health issues” increased to 38.1% (n = 8), mainly due to HIV infection while 14.3% (n = 3) claimed “Financial issues” and 4.8% (n = 1) “Professional/work-related issues.”

### 3.5 Use of assisted reproduction techniques

Only 4 of the 347 pregnancies occurred through assisted reproduction techniques and 3 took place after the HIV diagnosis of the women. The techniques undergone were artificial fertilization with intracytoplasmic injection for the pregnancy before HIV diagnosis (2001), and 2 artificial inseminations (2008 and 2009) and 1 in vitro fertilization with donated oocytes for the pregnancies after HIV diagnosis (2011). In 1 of these 4 cases sperm washing was performed due to HIV/HCV co-infection.

### 3.6 Antiretroviral therapy during pregnancy and HIV vertical transmission to the newborn

Of 101 pregnancies after HIV diagnosis, 61.4% (n = 62) were pregnancies in women receiving antiretroviral treatment. Of the 39 pregnancies in which women did not receive antiretroviral treatment, 31 (79.5%) ended in miscarriages or voluntary terminations, and in 4 women (10.2%) HIV diagnoses were during the last trimester of pregnancy or childbirth and in the remaining cases there was no information about this.

Of the 56 pregnancies to term in women with HIV infection, 3 children were infected with HIV, which gives a prevalence of 5.4%. These 3 cases of vertical transmission occurred in 1988, 1993, and 2004. In the cases of the children born in 1988 and 2004, the HIV diagnosis in the mother was made during labor. And in the case of the child born in 1993, the mother was diagnosed during the last pregnancy trimester follow-up. Neither of the mothers had received antiretroviral treatment. Otherwise, there was no vertical transmission among those mothers who received antiretroviral therapy even though when they were diagnosed during the prenatal follow-up.

The child born in 1988 did not receive antiretroviral treatment while the child born in 1993 did; in the case of the child born in 2004, the mother did not recall if the baby had received treatment or not. The remainder of the children received antiretroviral prophylaxis at birth.

In the 145 pregnancies to term in women before HIV diagnosis, 2 children were diagnosed with HIV infection retrospectively after their mother's diagnosis, at the age of 6 and 2 years. Pregnancy and childbirth of these 2 women occurred at their home countries, Ecuador, and Paraguay, while the diagnosis of HIV infection occurred when residing in Spain and attending health services due to health issues.

### 3.7 Factors associated with having pregnancies after HIV diagnosis

Table 3 shows the results of the bivariate and multivariable analyses of factors associated with having had a pregnancy after HIV diagnosis versus not having a pregnancy or having it before HIV diagnosis.

**Table 3:**
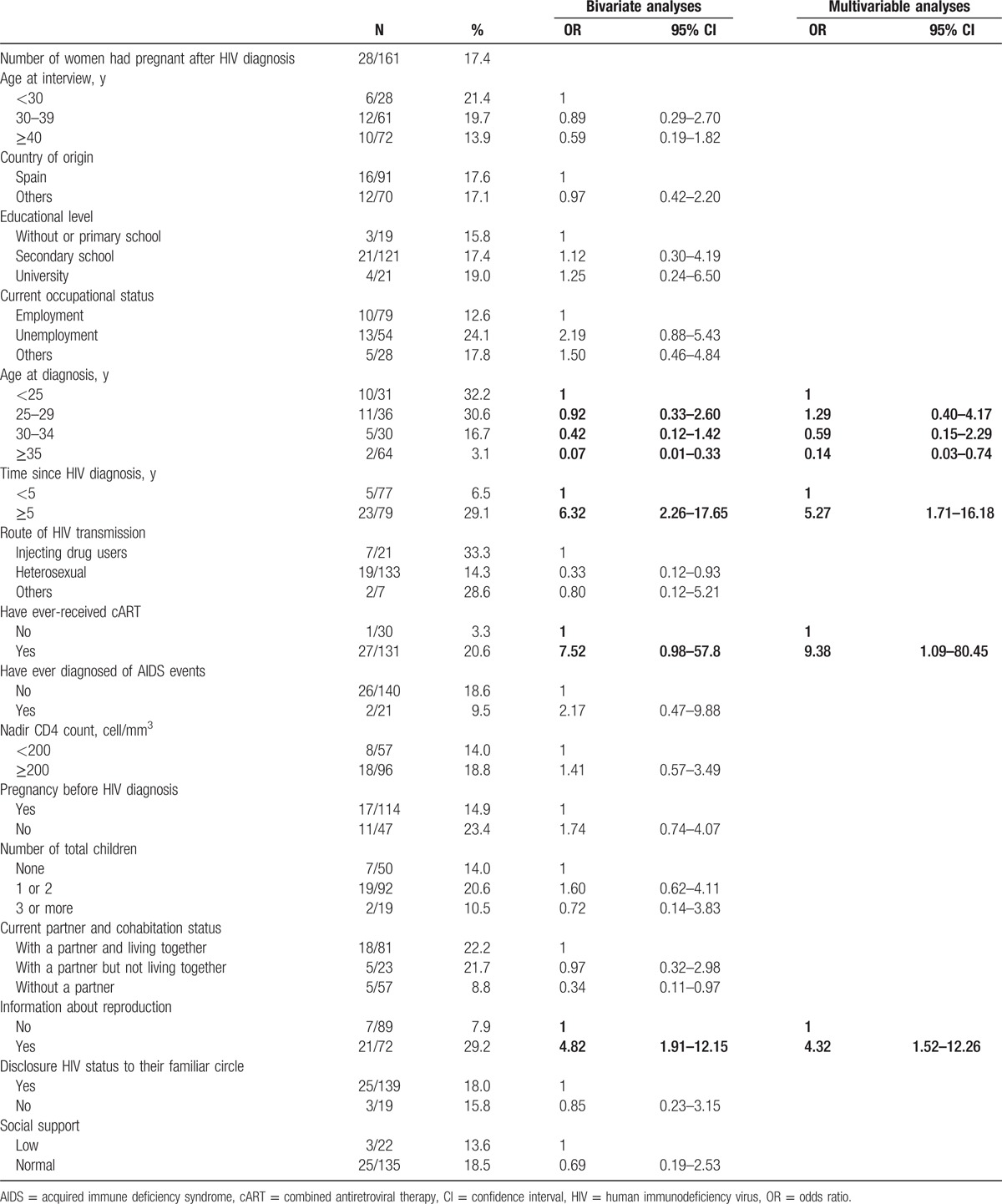
ORs for the association between becoming pregnant after HIV diagnosis and sociodemographic, clinical, and sexual behavior variables.

Of the 161 women interviewed, 28 (17.4%) had a pregnancy after HIV diagnosis, compared with 133 (82.6%) who either had no pregnancy or only had pregnancies before diagnosis (including HIV diagnosis during pregnancy or delivery).

In the multivariate analysis, being pregnant after diagnosis was associated with being younger at the time of diagnosis, OR = 1.29 (95% CI 0.40–4.17) for those ages 25 to 29 years, OR = 0.59 (95% CI 0.15 to 2.29) for those between 30 and 34 years, OR = 0.14 (95% CI 0.03–0.74) for those who were older than 35 years, compared to those with <25 years at diagnosis. It was also associated with having been diagnosed for 5 or more years, (OR = 5.27, 95% CI 1.71–16.18), having received antiretroviral therapy at some point (OR = 9.38, 95% CI 1.09–80.45), and having received information on reproductive health (OR = 4.32, 95% CI 1.52–12.26).

## 4 Discussion

Most of the women interviewed, 85.7% had been pregnant at least once in their life and 17.4% had a pregnancy after HIV diagnosis, accounting for 29.1% of all pregnancies. This percentage of women who had become pregnant after receiving their diagnosis is lower than that found in studies in Europe and United States where the percentage is between 25% and 35%.^[5,11,12]^

In our study, 21.7% of the women were diagnosed with HIV during pregnancy follow-up visits. In 3 of them, HIV diagnosis was made during labor. This highlights the importance of detecting cases through antenatal screening in women who do not feel at risk of acquiring the infection. Spain has adopted an opt-out strategy for HIV screening in pregnant women and recommends repetition of the test in the last trimester. If the woman is unaware of her HIV status at the time of childbirth, a rapid test is done to detect HIV antibodies and put in place preventive measures in case of a positive result.^[13]^ On the other hand, the cases detected during labor indicate the existence of important gaps in the health system that allow women to arrive to childbirth without any antenatal monitoring. Compared with women diagnosed during pregnancy follow-up, diagnosis at birth is clearly a missed opportunity for early diagnosis of HIV infection, which affects the mother and the newborn, and increases the risk of vertical transmission. In our study, the 3 children who were diagnosed with HIV were from mothers diagnosed during childbirth.

As for the pregnancies to term, no differences between pregnancies occurring before and after diagnoses were observed. Similarly, Massad et al^[14]^ observed that HIV-positive women had fewer pregnancies than HIV-negative ones, but found no differences in pregnancy outcome with respect to prematurity or birth weight.

We observed a rise in caesarean section deliveries in HIV-positive women. Caesarean section delivery has been one of the main guideline recommendations to prevent vertical transmission together with giving antiretroviral therapy to pregnant women and newborns, and avoiding breastfeeding. However, in recent years, several studies have shown that opting for a vaginal delivery when the woman is under antiretroviral therapy and the viral load is suppressed is an adequate alternative. The vertical transmission rate observed among women taking antiretroviral treatment is similar if the delivery is vaginal or through cesarean section^[15–17]^ and a study by the European Collaborative Study observed an increased risk of MTCT in women who had an inadequate control of their antenatal antiretroviral therapy.^[18]^ Therefore at this point we consider adherence to antiretroviral treatment in pregnant women with HIV infection to be paramount.

The diagnosis of HIV infection is a turning point in the sexual lives of these women, causing changes in their relationships and the use of contraceptive methods. Among the women who tested positive for HIV, 10.6% stopped having sex and the percentage of women who did not use contraception during sex decreased while the use of condom significantly increased.^[19]^ However, a substantial number of VTPs was observed after HIV diagnosis, possibly indicating that many of these pregnancies were unplanned. The percentage of VTPs after HIV diagnosis was 21.8%, similar to what we have seen in other recent studies in Europe^[5,7]^ and the United States.^[13]^ Although it is still an important percentage it is far below what was appreciated in the first decade of the epidemic and before the existence of combination antiretroviral therapy, when the incidence of pregnancy was significantly lower and VTP was very high after HIV diagnosis.^[3,20]^

It is worth noting the reasons given for VTP by women before and after HIV diagnosis. Before being diagnosed with HIV “Personal issues” was very frequent, especially being too young, interference with studies/career, the lack of stability in the relationship and the active use of drugs. After diagnosis, although HIV infection by itself was an important cause for VTP, “Personal issues” remained the main reason. In a study in Brazilian women with HIV infection, in addition to the fear of HIV transmission, the number of previous children, financial issues and the influence of family or medical opinion influenced the decision to terminate the pregnancy.^[21]^ Overall, these results show that information on use of contraception and reproductive health is lacking both in seropositive and seronegative women, suggesting that family planning services should improve. An unplanned pregnancy increases the risk of HIV transmission to the newborn and the probability of VTP.^[20,22]^

The factors associated with having a pregnancy after diagnosis were being diagnosed at a younger age, having received antiretroviral therapy at some point, and having received or sought information on sexual and reproductive health. Age at diagnosis was the most important factor implied in the desire for procreation and pregnancy in these women.^[5,11,23]^ The existence of combined antiretroviral therapy was also an incentive for women to go through motherhood. Improvements in antiretroviral treatment efficacy increased the number of pregnancies among HIV-positive women and the decline in abortions could be related to women's knowledge of a lower risk of transmission to the baby, along with a better perception of their health.^[24]^

Consulting a doctor or seeking information on reproductive health was also associated with having pregnancies after diagnosis. The involvement of health workers in giving reproductive, contraception, and sexual advice is essential for these women to normalize their sexual and emotional life. Many women would appreciate receiving advice from their physician on reproductive health, however, this rarely occurs,^[25,26]^ even when proof exists of it declining unwanted pregnancies and therefore the number of abortions.^[22]^

This study has a number of limitations. First, the sample size was small, mainly due to an important number of women who could not be located or who were not being followed up when the study was conducted. This was especially relevant in the largest hospitals (Madrid and Valencia), which are reference centers for HIV. It is easier for a patient to seek care in the initial phase after diagnosis and then be referred to another center closer to their home. However, no significant differences were observed between the women who were and were not interviewed (data not shown). Second, information about pregnancies was collected retrospectively and self-reported, so our results might be affected by recall bias, especially among first pregnancies and those before HIV diagnosis. The ad hoc questionnaire was carefully designed by the working group with a special focus in reducing the impact of this bias. Besides, the interviewers were trained to create a trustful atmosphere during the interviews.

Although our sample size was low, this study was nested within a cohort of patients recruited with high representation of the situation of the HIV epidemic in Spain which makes us believe this study provides a description of reproductive history and aspirations of women living with HIV in Spain.

In conclusion, the number of pregnancies after HIV diagnosis is considerable and reflects a clear desire for motherhood in HIV-positive women. Nevertheless the number of VTP is still high, indicating the need to reinforce the prevention of unplanned pregnancies and ameliorate the correct and timely delivery of information on sexual and reproductive health in HIV-positive women.

## Acknowledgments

This study would not have been possible without the collaboration of all the patients, medical and nursing staff, and data mangers who have taken part in the project.

## Supplementary Material

Supplemental Digital Content

## Supplementary Material

Supplemental Digital Content
