# Supplementary material for: Reproductive history before and after HIV diagnosis: A cross-sectional study in HIV-positive women in Spain
Source: Medicine (Baltimore). 2017 Feb 3;96(5):e5991. doi: 10.1097/MD.0000000000005991 (PMC5293456; doi:10.1097/MD.0000000000005991)
Supplement: Supplemental Digital Content [file medi-96-e5991-s001.doc]

**Annex 1: Centers and researchers involved in CoRIS:**

**Executive committe**: Santiago Moreno, Julia del Amo, David Dalmau, Maria Luisa Navarro, Maria Isabel González-Tomé, Federico García, José Luis Blanco, Rafael Rubio, Jose Antonio Iribarren, Francesc Vidal, Félix Gutiérrez, Juan Berenguer, Juan González.

**Fieldwork, data management and analysis**: Paz Sobrino Vegas, Victoria Hernando Sebastián, Belén Alejos Ferreras, Débora Álvarez, Yaiza Rivero, Inmaculada Jarrín, Cristina González.

**BioBanco:** M Ángeles Muñoz-Fernández, Isabel García-Merino, Coral Gómez Rico, Jorge Gallego de la Fuente y Almudena García Torre.

**Participanting centres:**

Hospital General Universitario de Alicante (Alicante): Joaquín Portilla, Esperanza Merino, Sergio Reus, Vicente Boix, Livia Giner, Carmen Gadea, Irene Portilla, Maria Pampliega, Marcos Díez, Juan Carlos Rodríguez, Jose Sánchez-Payá.

Hospital Donostia (San Sebastián): José Antonio Iribarren, Julio Arrizabalaga, María José Aramburu, Xabier Camino, Francisco Rodríguez-Arrondo, Miguel Ángel von Wichmann, Lidia Pascual Tomé, Miguel Ángel Goenaga, Mª Jesús Bustinduy, Harkaitz Azkune Galparsoro, Maialen Ibarguren, Miriam Aguado.

Hospital General Universitario de Elche (Elche): Félix Gutiérrez, Mar Masiá, Cristina López, Sergio Padilla, Andrés Navarro, Fernando Montolio, Catalina Robledano, Joan Gregori Colomé, Araceli Adsuar, Rafael Pascual, Federico Carlos, Maravillas Martínez.

Hospital Universitario La Fe (Valencia): Marta Montero, José López Aldeguer, Marino Blanes, José Lacruz, Miguel Salavert, Eva Calabuig, Sandra Cuéllar.

Hospital de la Princesa (Madrid): Ignacio de los Santos, Jesús Sanz, Ana Salas, Cristina Sarriá, Ana Gómez.

Hospital San Pedro-CIBIR (Logroño): José Antonio Oteo, José Ramón Blanco, Valvanera Ibarra, Luis Metola, Mercedes Sanz, Laura Pérez-Martínez

Hospital Ramón y Cajal (Madrid): Santiago Moreno, José Luis Casado, Fernando Dronda, Ana Moreno, María Jesús Pérez Elías, Dolores López, Carolina Gutiérrez, Beatriz Hernández, Nadia Madrid, Angel Lamas, Paloma Martí, Alberto de Diaz, Sergio Serrano, Lucas Donat.

Centro Sanitario Sandoval (Madrid): Jorge Del Romero Guerrero, Carmen Rodríguez Martín, Teresa Puerta López, Juan Carlos Carrió Montiel, Mar Vera.
